# Evaluating the effectiveness of sexual and reproductive health services during humanitarian crises: A systematic review

**DOI:** 10.1371/journal.pone.0199300

**Published:** 2018-07-06

**Authors:** Neha S. Singh, James Smith, Sarindi Aryasinghe, Rajat Khosla, Lale Say, Karl Blanchet

**Affiliations:** 1 Health in Humanitarian Crises Centre, London School of Hygiene & Tropical Medicine, London, United Kingdom; 2 Centre for Maternal, Adolescent, Reproductive and Child Health (MARCH), London School of Hygiene & Tropical Medicine, London, United Kingdom; 3 Department of Reproductive Health and Research, World Health Organization, Geneva, Switzerland; Ghent University, BELGIUM

## Abstract

**Background:**

An estimated 32 million women and girls of reproductive age living in emergency situations, all of whom require sexual and reproductive health (SRH) information and services. This systematic review assessed the effect of SRH interventions, including the Minimum Initial Service Package (MISP) on a range of health outcomes from the onset of emergencies.

**Methods and findings:**

We searched EMBASE, Global Health, MEDLINE and PsychINFO databases from January 1, 1980 to April 10, 2017. This review was registered with the PROSPERO database with identifier number CRD42017082102. We found 29 studies meet the inclusion criteria. We found high quality evidence to support the effectiveness of specific SRH interventions, such as home visits and peer-led educational and counselling, training of lower-level health care providers, community health workers (CHWs) to promote SRH services, a three-tiered network of health workers providing reproductive and maternal health services, integration of HIV and SRH services, and men’s discussion groups for reducing intimate partner violence. We found moderate quality evidence to support transport-based referral systems, community-based SRH education, CHW delivery of injectable contraceptives, wider literacy programmes, and birth preparedness interventions. No studies reported interventions related to fistulae, and only one study focused on abortion services.

**Conclusions:**

Despite increased attention to SRH in humanitarian crises, the sector has made little progress in advancing the evidence base for the effectiveness of SRH interventions, including the MISP, in crisis settings. A greater quantity and quality of more timely research is needed to ascertain the effectiveness of delivering SRH interventions in a variety of humanitarian crises.

## Introduction

The World Health Organisation defines health emergencies as sudden-onset events from naturally occurring or man-made hazards, or gradually deteriorating situations through which the risk to public health steadily increases over time.[1] It is estimated that 1 billion, or about 14% of the world’s population, live in areas affected by conflict.[2] According to the United Nations High Commissioner for Refugees, the number of forcibly displaced people has nearly doubled in the past two decades (from 33.9 million in 1997 to 65.6 million in 2016), with numbers remaining at a record high.[3] Given the changing nature of conflict and protracted crises, the average time spent in displacement has now reached 20 years.[3] The United Nations Population Fund estimates that of the more than 100 million people in need of humanitarian assistance in 2015, 32 million were women and girls aged 15–49 years.[4]

Women and girls are disproportionately affected in both sudden and protracted emergencies,[5] and face multiple sexual and reproductive health (SRH) challenges in conflict and post-conflict settings.[6] Inadequate or interrupted access to SRH services can also increase the number of people affected, generating a high risk of: mortality or morbidity due to pregnancy-related causes; unintended or unwanted pregnancies due to lack of information or access to contraceptive services; complications related to unsafe abortions; sexual and gender-based violence (SGBV); and an increased incidence of sexually transmitted infections (STIs), including HIV.[7, 8] These challenges limit women’s empowerment and their participation in the recovery process, resulting in violations of their human rights, and a reduction in the resources available to alleviate suffering and to be directed towards the process of recovery. Furthermore, there is an economic case for investing in SRH services in humanitarian crises settings, which face financial and other constraints, as it is estimated that for each additional $1 US dollar spent on contraceptive services above the current level of funding in crisis settings, the cost of maternal and newborn health-related care would be reduced by $2.22.[4]

Over the past two decades, there has been increased attention to SRH in populations in humanitarian settings. In 1994, the importance of refugees’ rights to SRH were articulated and globally agreed in Chapter X of the proceedings of the International Conference on Population and Development in Cairo.[9] In 1995, the growing awareness of, and commitment to, addressing emergency SRH needs culminated in the formation of the Inter-Agency Working Group (IAWG) on Reproductive Health in Crises, tasked with promoting access to quality SRH care among women and other vulnerable populations impacted by humanitarian crises.[10] In 1999, the IAWG developed a field manual to provide guidance to field staff on reproductive health interventions in emergencies, and included a chapter on the Minimum Initial Service Package (MISP), to be implemented within 48 hours of the onset of every humanitarian crisis. The MISP aims to facilitate the coordination of SRH services, prevent and manage the consequences of sexual violence, reduce HIV transmission, minimise maternal and neonatal morbidity and mortality, and plan for comprehensive SRH services in the post-crisis phase.

Despite increasing attention to SRH in humanitarian settings, recent evaluations of the MISP have been mixed.[11, 12] A systematic review in 2015 [13] found no peer-reviewed papers that evaluated MISP implementation comprehensively since the first global evaluation in 2004, which identified a low awareness among health actors, and no systematic implementation of the MISP.[14] The literature is even sparser for studies assessing the effectiveness of SRH interventions including the MISP in humanitarian crises.[13, 15, 16] The only systematic review conducted to date to assess the effectiveness of SRH interventions in humanitarian settings reported a low quantity and quality of evidence, but notably did not comprehensively search for studies focusing on abortion, or aim to disaggregate data by vulnerable sub-populations, e.g. adolescents.[15]

To build upon progress made and to address evidence gaps, we aimed to consolidate the existing evidence base for the effectiveness of SRH interventions including the MISP from the onset of emergencies by conducting a broader systematic review. Our systematic review aimed to assess the effect of SRH interventions including the MISP and its components reported in the peer-reviewed literature between 1980 and 2017 on a range of health outcomes from the onset of emergencies. As part of this objective, we also aimed to assess issues related to targeting of SRH interventions including the MISP, e.g. whether they are delivered to vulnerable populations such as those with disabilities, sex workers, adolescent girls, and lesbian, gay, transsexual, queer and intersex (LGBTQI) populations.

## Methods

This systematic literature review adhered to the Preferred Reporting Items for Systematic Reviews and Meta-Analyses (PRISMA) statement (**S1 Appendix**).[17] The review was registered with the PROSPERO database with identifier number CRD42017082102. The inclusion and exclusion criteria are detailed in Table 1.

**Table 1:** Inclusion and exclusion criteria.

Category	Included	Excluded
Population of interest	Crisis-affected populations receiving humanitarian assistance or aid in low-income or middle-income countries (as defined by World Bank, 2012): including refugees and internally displaced persons	
Intervention	Any health-related intervention seeking to improve SRH outcomes	
Outcomes of interest	Primary outcomes include adolescent, maternal and neonatal morbidity; adolescent, maternal and neonatal mortality; STI diagnosis; gender-based violence; and unmet need for family planning. Secondary outcomes include contraceptive prevalence rate; skilled attendance at birth; and emergency obstetric and newborn care (EmONC)	- Studies which do not quantify health outcomes - Studies only measuring knowledge, attitudes and practice as outcomes
Study types and design	Primary quantitative research studies. Study designs including randomised controlled trials, non-randomised controlled trials, controlled before-after studies, controlled interrupted time series studies, economic studies (cost-effectiveness analysis, cost-utility analysis, cost-benefit analysis, economic modelling) of public health which the outcome is measured before and after the intervention or an intervention is studied against another intervention with baseline or control group.	- Studies on preparedness or resilience if not linked to an intervention evaluating the effectiveness or utilisation of SRH interventions - Studies only targeting child sexual abuse - Studies with no specific health intervention and no outcomes (i.e. studies examining only health needs, prevalence, health risk-factors, co-ordination). - Review papers; references listed in review papers will be screened to find more primary data sources
Data type	Must include primary data	
Phase of humanitarian crises	Studies conducted during the acute, chronic and early recovery phases of a humanitarian crisis	
Publication date	January 1, 1980 –April 10, 2017	
Language	English, French	Other languages

### Search strategy and selection criteria

Search terms for SRH were based on the definition of SRH from the International Conference on Population and Development in 1994 [9] and from the World Health Organisation’s SRH strategies and guidance in 2010 [18] and 2017 [19]. SRH refers to a state of physical, emotional, mental and social well-being in relation to sexuality and reproductive health; it is not merely the absence of disease, dysfunction or infirmity. [4, 18] International guidelines on SRH in conflict-affected settings include activities related to family planning, abortion, HIV/AIDS and STIs including PMTCT, maternal and newborn health, and SGBV.[20]

We defined a humanitarian crisis as a serious disruption of the functioning of a community or a society causing widespread human, material, economic or environmental losses that exceed the ability of the affected community or society to cope using its own resources, necessitating a request to the national or international level for external assistance. The crisis situation may either be man-made (e.g. armed conflict) or a natural phenomenon (e.g. drought). Only studies from low-income or middle-income countries were included in this study, as the majority of humanitarian crises occur in these countries, and the resources and strategies available to address them are very different in high-income countries.

A detailed protocol with specific search terms are provided in S2 Appendix, and were generated by the authors and then supplemented by searching for other search strategies used in previous systematic reviews on similar topics.[21, 22] We also consulted a trained information science and Cochrane review specialist at the London School of Hygiene and Tropical Medicine to review our literature searching syntax and strategy.

We included studies from peer-reviewed journals across the following four databases: EMBASE, Global Health, MEDLINE and PsychINFO. We complemented searches by screening the reference lists of papers for potentially relevant studies, as well as reference lists of relevant systematic reviews. We also consulted experts on SRH service delivery and research to identify additional research not identified during our systematic search (see Acknowledgments section for list of experts). Selection criteria for literature are detailed in Table 1.

### Data analysis

**Fig 1:**
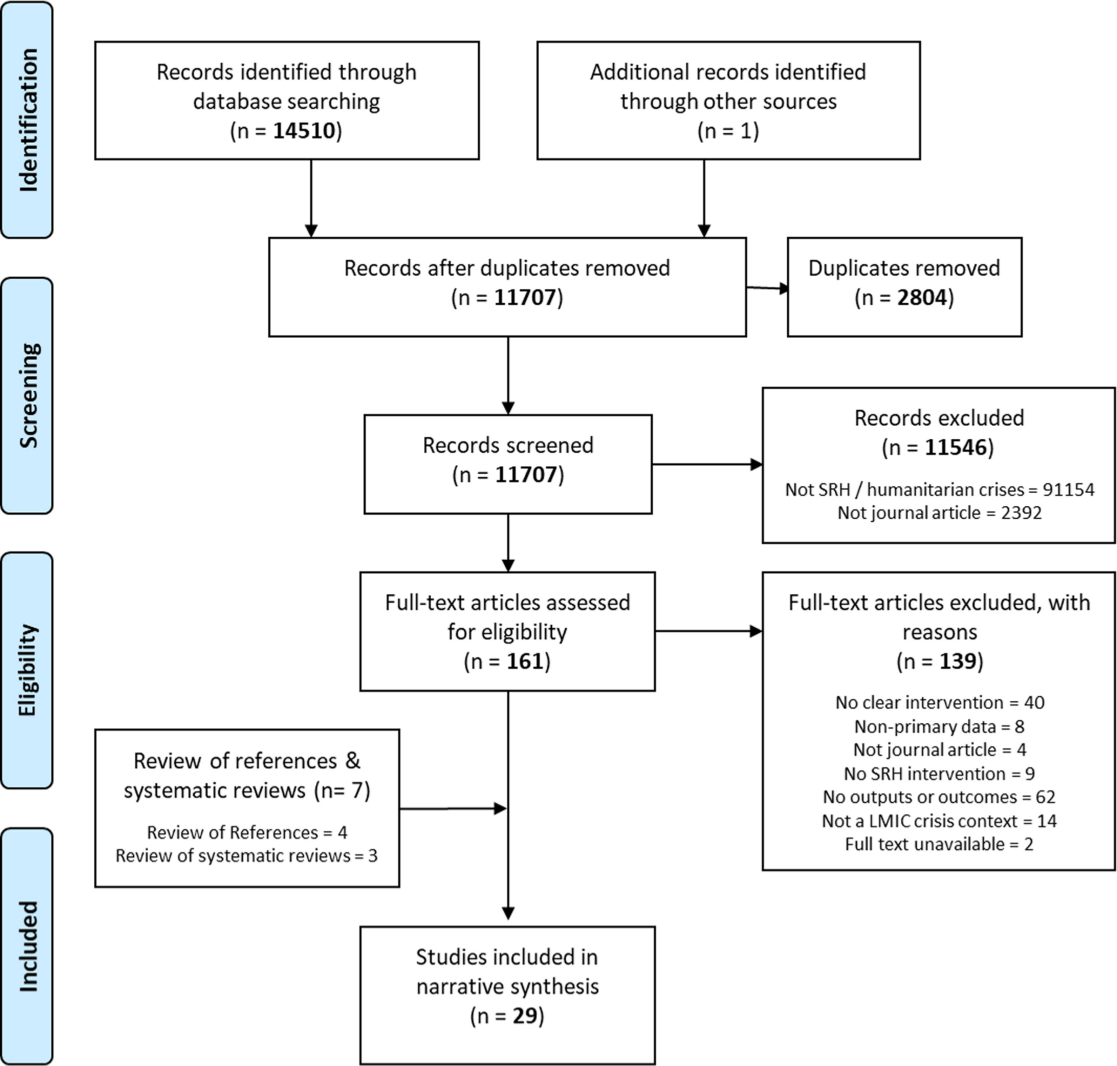
Selection process for systematic review on the effectiveness of SRH interventions in humanitarian crises settings.

We downloaded all returned citations from the searched databases into an Endnote library and applied a standard data-screening process (Fig 1). We based the primary and secondary outcomes of interest for inclusion from the IAWG field manual,^14^ as it is an established and widely used manual for SRH in crisis-affected settings, and was selected based on discussion with the SRH expert committee. Inclusion and exclusion criteria applied during screening are outlined in Table 1. Data from the final selected studies were then extracted into a Microsoft Excel database, with data extraction fields including study author and year, setting, crisis type, crisis stage, study design and methods, research setting, health outcomes and intervention descriptions. First round data screening and extraction were independently conducted by NSS, JS and SA. Second round detailed data extraction was conducted independently and in duplicate by NSS and JS.

We used a narrative synthesis approach due to the heterogeneity of study outcomes, interventions and methods. Findings were synthesised by main SRH outcomes including those included in an earlier systematic review by Warren et al. on SRH interventions in humanitarian settings [15] i.e. family planning; abortion; prevention, treatment, and care for STIs including HIV/AIDS; maternal, newborn and child health, including obstetric care; SGBV; and studies with cross-cutting SRH themes. These themes were developed iteratively after thematic analysis of the studies’ stated aims and primary reported health outcome of interest.

The quality of reporting in the included studies were assessed using the Strengthening the Reporting of Observational Studies in Epidemiology (STROBE) and Consolidated Standards of Reporting Trials (CONSORT) checklists,[23, 24] which are commonly used for reporting the quality of observational studies and randomised controlled trials. Both checklists include measures regarding the reporting of participant selection, sample sizes, variables, data sources, bias, descriptive and outcome data, interpretation, and generalisability, among others. In order to further explore the quality of reporting, we awarded papers one point for reporting each of the items on the relevant checklist. When totalled, these points formed the numerator of a proportional score, with the denominator the total number of possible relevant checklist items which varied slightly by study type, i.e. 22 total points for the STROBE checklist, and 25 total points for the CONSORT checklist. NSS, JS and SA conducted quality assessment, who each independently evaluated the quality of all included studies and discussed each discrepancy until consensus was reached. For this systematic review, the study team determined a priori that papers with a score of <33% were considered low reporting quality, moderate quality if 34–66%, and high quality if >67%. These quality thresholds have been used in a previous systematic review on SRH in humanitarian crises settings.[22]

## Results

A total of 14,510 citations were returned from peer-reviewed databases, with one additional study provided by expert recommendation (**Fig 1**). Following full screening, the review of reference lists, and a review of two existing systematic reviews on similar topics, [13, 15] a total of 29 studies met the inclusion criteria (**Table 2**).

**Table 2:** Study characteristics and key findings.

Author (year)	Study country	Setting	Crisis type	Crisis stage	Target population	Intervention	Study design	Key findings	Quality
**Family planning**									
Howard et al. (2008)	Guinea	Camp	Armed Conflict	Stabilised	Refugee	Development of a refugee led “Reproductive Health Group”	Cross-sectional	Those who reported RHG facilitators as their primary information source had non-significantly higher odds of being current users of contraception (OR = 1.3, 0.7–2.6, adjusted for parity, education, and partner approval of FP)	High
Huber et al. (2010)	Afghanistan	Rural	Armed Conflict	Chronic	General Population	Health education, CHW delivery of injectable contraceptives	Pre-Post Study	The REACH Project achieved an increase of contraceptive use from 16% to 26%, over a period of 2 years in 13 provinces. The ACU Project increased contraceptive use by 24–27% in its three sites over 8 months	Moderate
Casey et al. (2013)	Uganda	Rural	Armed Conflict	Stabilised	IDP / General Population	Mobile outreach and public health centre strengthening	Cross-sectional	Increased ever use of FP method 27.6% [23.5–32.2] to 47.3% [43.6–51.1], (aOR 2.23 [1.7–2.92] p<0.001). Unmet FP need 52.1% [48.5–55.6] to 35.7% [32–39.6], (aOR 0.47 [0.37–0.6], p<0.001)	High
Curry et al. (2015)	Multi-Country (Chad, DRC, Djibouti, Mali, Pakistan)	Urban / Rural	Armed Conflict / Natural Disaster	Acute	IDP / Refugee / General Population	Training, facility supervision, supply of contraceptives, community mobilisation and awareness raising	Cross-sectional	Increase in new modern FP method users over time, notably for new users choosing long-acting and reversible contraceptives (78% in the DRC, 72% in Chad, and 51% in Mali, 29% in Pakistan).	Moderate
Adam (2016)	Sudan	Camp	Armed Conflict	Chronic	IDP	Home counselling and awareness raising	Cross-sectional	Increased use of modern family planning methods (aOR 2.8, 95%CI 2.0–4.1)	High
Raheel et al. (2012)	Pakistan	Urban	Armed conflict	Stabilised	Refugee	Subsidised healthcare (90% subsidies for doctor's visits, hospital visits, emergency care, free family planning, excluding prescriptions)	Cross-sectional	Reported use of contraceptives in subsidised group (54%) was more than double the use reported in the non-subsidised group (25%), (P<0.001); non-subsidised group more likely to use the pill (40.7%), subsidised group more likely to have tubal ligation (36.7%), p<0.001.	High
**PMTCT**									
Bannick-Mbazzi (2013)	Uganda	Rural	Armed Conflict	Chronic	General Population	Comprehensive PMTCT programme	Cross-sectional	Between 2004 and 2011, prevalence of HIV in children 6 weeks—18 months old declined from 10.3% to 5% (p = 0.01). Increase in number of HIV positive women delivering in a health facility (56% to 81%, p-0.033)	High
**HIV/Sexually transmitted infections (STIs)**									
Larsen et al. (2004)	Sierra Leone	Urban	Armed Conflict	Chronic	General Population	AIDS prevention programme–community outreach and education	Pre-Post Study	At post-intervention, 68 per cent of CSWs reported using a condom at their last sexual encounter as compared to only 38 per cent at baseline. At post-intervention 83 per cent reported having ever used a condom, as compared to 60 per cent at baseline. At post-intervention, 82 per cent of military respondents reported having ever used a condom up from 66 per cent in 2001, while the proportion of those who reported using a condom at last sexual intercourse increased from 39 per cent to 68 per cent of respondents.	High
Casey et al. (2006)	Sierra Leone	Urban	Armed Conflict	Chronic	IDP / General Population	HIV prevention activities	Pre-Post Study	At baseline, fewer than one in five (15.6%) female youth reported condom use the last time they had sex, while nearly half (46.2%) reported this at post-intervention. Similarly, only one in four (24.8%) reported having ever used a condom at baseline as compared to nearly two in three (63.6%) at post-intervention. The proportion of male youth reporting having used a condom the last time they had sexual intercourse increased from 15.6% at baseline to 37.1%. While one in four (26.4%) respondents reported having ever used a condom at baseline, one in two (50.2%) reported having ever used a condom at post-intervention.	High
Culbert et al. (2007)	DRC	Urban	Armed Conflict	Chronic	General Population	Initiation of anti-retroviral treatment	Cohort	6 month median weight gain 2.5kg (0–5.5), 6 month medial CD4 gain 163 (82–232), 12 month mortality 7.9% (3.6%-12.1%), 12 month LTFU 5.4% (3.2–7.5)	Moderate
O’Brien et al. (2010)	Ten sub-Saharan African countries, Colombia, India	Urban, Rural & Camp	Armed Conflict & Natural Disaster	Acute & Chronic	IDP / Refugees / General Population	HIV service integration	Cross-sectional	Median 12-month survival of 0.89 (95% CI 0.88–0.91) and a median 6-month CD4 gain of 129 cells/mm^3^ following the integration of HIV care and treatment programmes with other medical activities	High
Logie et al. (2014)	Haiti	Camp	Natural Disaster	Chronic	IDP	Weekly psycho-educational and Peer Health Worker-led psycho-educational HIV-STI prevention	Cohort	Increase in condom use (AOR 4.05, 95% CI: 1.86, 8.83, p<0.001)	High
**Pregnancy & maternal and newborn health**									
Samai & Sengeh (1997)	Sierra Leone	Urban	Armed Conflict	Acute	General Population	Investments in vehicular referral system, community education, health facility improvements	Pre-Post Study	Service utilisation more than doubled in the period following initiation of the transport system. The case fatality rate declined from 20% to 10% in the post-intervention period.	Moderate
McPherson et al. (2006)	Nepal	Urban & Rural	Armed Conflict	Stabilised	General Population	Community education, birth preparedness programme	Pre-Post Study	The proportion of women reporting one or more antenatal care visit increased from 60% to 84% (p<0.001), and use of postnatal care within six weeks of delivery increased from 45% to 72% (p<0.001). Changes in the use of a skilled birth provider were not statistically significant.	High
Hadi et al. (2007)	Afghanistan	Rural	Armed Conflict	Chronic	General Population	Introduction of a community-based safe motherhood programme	Pre-Post Study	Pregnant women reached by CHW—40.3% in 2004 to 95.5% in 2006 (p<0.01); received antenatal care—37.3% in 2004 to 91.2% in 2006 (p<0.01); institutional delivery 31.3% to 55.2% (p<0.01)	High
Purdin et al. (2009)	Pakistan	Urban & Rural	Armed Conflict	Chronic	Refugee	Establishing emergency obstetric care (EmOC) centres, community training on safe motherhood, linking primary health care with education on pregnancy danger signs and importance of skilled attendance at birth, improving health information system	Cross-sectional	The proportion of refugee births in an EmOC facility increased from 4.8% in 1996 to 67.2% in 2007. MMR reduced from 291 to 102 per 100,000 live births from 1st to 5th year of programme (95% CI 181 to 400); NMR reduced from 25 to 20.7 per 1000 live births from 1st to 7th year.	Moderate
Turner et al. (2013)	Thailand	Camp	Armed Conflict	Chronic	Refugee	Development of a Special Care Baby Unit and associated training	Cross-sectional	NMR decreased from 21.8 deaths per 1000 live births to 10.7 deaths per 1000 live births (p = 0.03) between 2008 and 2011. Cause specific mortality fell in all of the four main causes of death overall: prematurity (19.3% to 4.8%), Early Onset Neonatal Sepsis (6.0% to 1.8%), congenital abnormality (60% to 22.2%) and jaundice (2.2% to 0.6%).	Moderate
Adam (2015)	Sudan	Camp	Armed Conflict	Chronic	IDP	Home-based maternal health education	Cross-sectional	Maternal health education reduced odds of home birth (aOR 0.57)	High
Adam et al. (2015)	Sudan	Camp	Armed Conflict	Chronic	IDP	Interpersonal communication and mass education campaigns	Cross-sectional	Education campaigns increased likelihood of at least 3 antenatal care visits (OR 8.8, 95% CI 6.4–12), healthcare-facility based delivery (OR 5.4, 95% CI 4.0–7.4), 1 or more postnatal care visits (OR 5.5, 95% CI 4.0–7.7).	High
Groppi et al. (2015)	South Sudan	Urban & Rural	Armed Conflict	Chronic	General Population	Ambulance-based referral system	Cross-sectional	Facility-based deliveries increased in 2012 to 1089 (13.3% of expected deliveries in catchment area). 38.3% of women in need of EmOC received such care. CS proportion 0.6%.	Moderate
Castillo et al. (2016)	Philippines	Urban & Rural	Natural Disaster	Early Recovery	General Population	Training of trainers and quality assessment workshops	Pre-Post Study	24/7 skilled birth attendance (approx. 84% to 96%), kangaroo mother care (approx. 41% to 94%).	High
Pham et al. (2016)	Sudan	Urban & Camp	Armed Conflict	Acute	IDP / Refugee	Staff training, primary healthcare service provision	Cross-sectional	Skilled birth attendance increased from 35.7% to 52.7% (p = 0.025)	High
**Sexual and gender-based violence**									
Gupta et al. (2013)	Ivory Coast	Rural	Armed Conflict	Acute	General Population	Gender dialogue groups, economic empowerment programme	RCT	VSLA + GDG less likely to report economic abuse than VSLA-only (OR 0.39, CI 0.25–0.6, p<0.0001); acceptance of justification towards violence was reduced (B = -0.97, CI -1.67, -0.28, p = 0.006). Highly adherent women in VSLA + GDG group less likely to report physical violence (aOR 0.45, CI 0.21–0.94, p = 0.04)	High
Hossain et al. (2013)	Ivory Coast	Rural	Armed Conflict	Acute	General Population	Men’s discussion group	RCT	Men more likely to use one positive conflict management technique (aRR 1.3, CI 1.06–1.58); men involved in at least two household tasks (aRR 2.47, CI 1.24–4.9).	High
Bass et al. (2013)	DRC	Rural	Armed Conflict	Chronic	General Population	Individual psychological support, group cognitive therapy	RCT	Individual support (HSCL-25 score 1.7+/-0.7 end of treatment, 1.5+/-0.6 6 months post-Tx; p<0.001; PTSD checklist 1.7+/-0.8 end of treatment, 1.5+/-0.7 6 months post-Tx, p<0.001; functional-impairment score 1.9+/-0.9 end of Tx, 1.8+/- 0.9 6 months post-Tx; p<0.001) and therapy groups (HSCL-25 score for depression and anxiety 0.8+/-0.6 end of treatment; 0.7+/-0.6 6 months post-Tx; p<0.001; PTSD checklist score end of treatment 0.8+/- 0.6 end of treatment, 0.7+/-0.6 6 months post-Tx, p<0.001; functional impairment score 0.8+/-0.07 end of Tx, 0.9+/-0.7 6 months post-Tx; p<0.0001) had significant improvements during treatment, with effects maintained at 6 months	High
**Interventions targeting outcomes across multiple SRH domains**									
Leigh et al. (1997)	Sierra Leone	Urban	Armed Conflict	Early Recovery	General Population	Skilled staff deployment, training, provision of supplies, enhanced community referral	Cross-sectional	The proportion of women accessing the hospital increased from 31 in 1990 to 98 in 1995, with a reduction in the case fatality rate from 32% to 5%. In addition, 444 abortion-related procedures were performed, compared with only 22 in 1990.	Moderate
McGinn & Allen (2006)	Guinea	Camp	Armed Conflict	Early Recovery	Refugee	Reproductive health literacy programme	Cross-sectional	50% of the survey respondents reported current use of modern contraceptives, while 24% reported using a condom the last time they had sex, of which both findings were interpreted as an increase since implementation of the reproductive health literacy programme. 92% of women who reported becoming pregnant since the reproductive health literacy programme reported attending at least three antenatal visits.	High
Mullany et al. (2010)	Myanmar	Rural	Armed Conflict	Chronic	IDP	Training of community-based healthcare providers, antenatal, obstetric, and family planning service provision	Cross-sectional	Use of a modern method of contraception increased from 23.9% to 45.0% (prevalence rate ratio (PRR) 1.88, 95% CI 1.63, 2.17). Unmet family planning needs dropped from 61.7% to 40.5% (PRR 0.65, 95% CI 0.60, 0.72), while birth attendance by someone trained in emergency obstetric care increased from 5.1% to 48.7% (PRR 9.55, 95% CI 7.21, 12.64).	High
Zaman et al. (2013)	Pakistan	Urban & Rural	Natural Disaster	Stabilised	General Population	Health system strengthening including strengthening management capacities of district health authorities, improving access to quality primary healthcare services, increasing participation of communities in health service management, and improving household level knowledge and care-seeking behaviours	Cross-sectional	Increases in the use of modern contraceptives (18% to 22%), at least one antenatal care visit (70.3% to 73.6%), and presence of a skilled birth attendant (36% to 38%) were non-significant. A statistically significant increase in receipt of postnatal care from 25% to 33.3% was reported (p<0.01).	High

Of the 29 included studies, 23 (79.3%) were published during the last decade between January 2007 and March 2017. In relation to the geographical distribution of the studies, 17 studies (58.6%) were conducted in Africa, a further nine studies (31.0%) in Asia, one study was conducted in Haiti (3.4%) while the remaining two studies (6.9%) both comprised sites in multiple countries. In relation to the typology of humanitarian crisis, 24 studies (82.8%) were conducted in areas affected by armed conflict, and the two multi-site studies (6.9%) were conducted in areas affected by both armed conflict and natural disasters. The remaining three studies (10.3%) were conducted in areas affected by a natural disaster: the first study focused on the 2005 earthquake in northern Pakistan; the second study focused on the 2013 Typhoon Haiyan in the Philippines; and the third study was conducted in the aftermath of the 2010 earthquake in Haiti.

Of the included studies, over a third (34.5%, n = 10) examined the effectiveness of interventions relating to pregnancy and maternal and newborn health (MNH). Six studies (20.7%) assessed family planning interventions, while a further five studies (17.2%) assessed interventions addressing either HIV or STIs. Three studies (10.3%) examined interventions related to SGBV, while only one study (3.4%) focused on the prevention of mother-to-child transmission of HIV (PMTCT). The remaining four studies (13.8%) assessed a number of cross-cutting SRH domains, of which three studies (10.3%) focused on both family planning and MNH interventions, and one study (3.4%) addressed family planning and abortion care. Notably, no studies addressed interventions aimed at responding to, or the prevention of, vaginal injuries and fistulae.

### Study quality

All 29 studies were assessed for quality of reporting using the STROBE checklist. The majority of the observational studies (72.4%, n = 21) were found to be of high quality,[25–44] and the remaining eight studies (27.6%) were of moderate quality.[45–51]

There were common areas in which the included studies in this review provided low quality reporting. All moderate quality quantitative studies stated changes in health outcomes, but statistical associations between the intervention and the outcome were inconsistently reported, i.e. most studies did not report differences between SRH interventions and outcomes. Second, it was also not clear whether relevant confounders and biases were considered during the design of the study and analysis of the data.

### Family planning

We identified six studies related to family planning. Along with other SRH outcomes, all six studies assessed contraceptive use in their respective contexts. One paper assessed a refugee-led reproductive health group operating across 48 Guinean refugee camps that recruited refugee nurses and midwives to local health facilities, trained lay women to provide health education and contraception, and to facilitate referrals. Individuals who reported the reproductive health group facilitators as their primary source of information were more likely to be current users of contraception (aOR = 1.3, 95% CI 0.7, 4.2, non-significant).[25] A home-based counselling and awareness programme for internally displaced women in Sudan led to an increase in the use of modern family planning methods (aOR 2.8, 95% CI 2.0, 4.1).[27] A programme of mobile outreach and public health strengthening in Uganda led to an increase in the number of women who reported ever using a family planning method (aOR 2.23, 95% CI 1.7, 2.92, P<0.001), and a reduction in the unmet need for family planning from 52.1% to 35.7% (aOR 0.47, 95% CI 0.37, 0.6, P<0.001).[26] A study in Pakistan providing subsidised healthcare to refugees reported use of contraceptives in the subsidised group (54%) was more than double the use reported in the non-subsidised group (25%), (P<0.001), and reported that the non-subsidised group was more likely to use the oral contraceptive pill (40.7%), whereas the subsidised group was more likely to have tubal ligation (36.7%) (p<0.001).[52] A multi-country study involving sites in Chad, the Democratic Republic of the Congo, Djibouti, Mali, and Pakistan assessed the impact of a CARE programme that included staff training, supervision in health facilities, the supply of contraceptives, community mobilisation, and awareness raising. An absolute number of 52,616 new users of modern methods of contraception were reported, of which an average of 61% of users across all sites opted for long-acting reversible contraception.[46] A study in rural Afghanistan assessed the impact of health education and the delivery of injectable contraceptives by community health workers. Over an eight-month period between 2005 and 2006, contraceptive use increased by 24–27% across three sites.[45]

### Pregnancy & maternal and newborn health

We included ten studies reporting on pregnancy or maternal and newborn health-related outcomes. A cross-sectional study amongst internally-displaced women in Darfur, Sudan assessed the impact of a maternal health education programme delivered in the form of home visits. Receiving maternal health education at home was associated with a 43% reduction in the odds of giving birth at home as compared to in a healthcare facility (aOR 0.57, 95% CI: 0.35, 0.93).[34] Another study amongst internally-displaced women in Darfur found that following the implementation of an interpersonal and mass education campaign, women were more likely to deliver at a healthcare facility (OR 5.4, 95% CI: 4.0, 7.4, P<0.001).[35] Two studies assessed the effect of referral systems on access to hospital-based care. Following the implementation of an ambulance-based referral system and improved hospital telecommunications in Yirol, South Sudan, the authors reported that 99.1% of the estimated number of women with absolute obstetric indications were treated at the hospital.[51] A second study examined the impact of a hospital-based vehicle, motorbikes at satellite primary healthcare centres, along with community education activities and facility improvements, on service utilisation and case fatality rates in Bo, Sierra Leone during the period 1992–1993. Service utilisation more than doubled in the period following initiation of the transport system. The case fatality rate declined from 20% to 10% in the post-intervention period. However, there was no significant difference in outcomes between those who were referred using a hospital vehicle and those who used other means to reach the hospital.[48]

In the Philippines, following Typhoon Haiyan, training of trainers and quality assurance workshops were held, with subsequent improvements in 24-hour access to skilled birth attendants from 84% to 96% at three months (p<0.05).[36] An evaluation of a programme in West Darfur, Sudan implemented by Medair, including primary healthcare service provision, health promotion activities, and the training of midwives, reported an increase in skilled birth attendance from 35.7% to 52.7% (p = 0.025).[37] The implementation of a community-based safe motherhood programme in Kabul Province, Afghanistan, delivered by a cadre of newly trained community midwives, reported an increase in the proportion of women receiving antenatal care from 37.3% in 2004 to 91.2% in 2006 (p<0.01), and an increase in the proportion of women delivering at a healthcare facility from 31.3% to 55.2% (p<0.01).[33]

An evaluation of an International Rescue Committee (IRC) programme, which aimed to reduce maternal mortality among Afghan refugees in Pakistan by establishing emergency obstetric care centres, training community members on safe motherhood, and linking primary care with pregnancy-related education, reported a reduction in the maternal mortality ratio from 291 to 102 per 100000 live births between the first and fifth years of the programme. A reduction in the neonatal mortality rate from 25 to 20.7 per 1000 live births was also reported during the same period, while the proportion of births registered in an emergency obstetric care facility increased from 4.8% in 1996 to 67.2% in 2007.[49] A study conducted by Save the Children evaluated a birth preparedness package programme implemented in eastern Nepal between 2003 and 2004. The programme involved an educational component delivered by community healthcare workers, and preparation in advance of delivery to optimise maternal and newborn health outcomes. Along with improvements in essential newborn practices, the proportion of women reporting one or more antenatal care visit increased from 60% to 84% (p<0.001), and use of postnatal care within six weeks of delivery increased from 45% to 72% (p<0.001). Changes in the use of a skilled birth attendant were not statistically significant.[32]

Another study conducted in the Maela refugee camp on the Thai-Myanmar border assessed the effect of a special care baby unit on neonatal and cause-specific mortality rates. Between 2008 and 2011, the neonatal mortality rate declined from 21.8 to 10.7 deaths per 1000 live births (p = 0.03), while cause-specific mortality also fell in relation to the four main causes of neonatal death: prematurity, early onset neonatal sepsis, congenital abnormalities, and jaundice.[50]

### HIV and STIs

A study conducted by Médecins Sans Frontières (MSF) in the conflict-affected region of Bukavu, eastern Democratic Republic of the Congo, reported a median weight gain of 2.5kg and a CD4 gain of 163 cells/ml at 6-months for patients initiated on generic, fixed dose anti-retroviral treatment.[47] A second study on 24 MSF programmes in 12 countries reported a median 12-month survival of 0.89 (95% CI: 0.88, 0.91) and a median 6-month CD4 gain of 129 cells/mm^3^ following the integration of HIV care and treatment programmes with other medical activities.[30]

A third non-randomised cohort study amongst internally displaced women in Haiti reported an increase in condom use (AOR 4.05, 95% CI: 1.86, 8.83, p<0.001) following implementation of a peer health worker-led intervention involving a video-based session on HIV and STIs, followed by a six-week psycho-educational programme.[31] A pre-post study of an HIV/AIDS and STI prevention project, including outreach and education activities, in Sierra Leone identified an increase from 38% condom use at last sexual encounter to 68% amongst commercial sex workers, and from 39% to 68% amongst those who identified as members of the military forces.[28] Following the roll-out of HIV prevention education activities in an urban setting in Sierra Leone amongst adolescents aged 15 to 24 years, contraceptive use increased from 16% to 46% amongst female adolescents, and from 16% to 37% amongst male adolescents (p < .01) [29].

### PMTCT

One study examined the effectiveness of a comprehensive PMTCT programme in conflict-affected northern Uganda. Over the course of a ten-year period, the proportion of HIV-positive women delivering in a health facility increased from 56% to 81% (p = 0.033).[44]

### Sexual and gender-based violence

Three studies, all of which were randomised controlled trials, reported SGBV outcomes. A trial in the Ivory Coast compared the impact of participation in a gender dialogue group and an economic empowerment programme, versus participation only in the economic empowerment programme. Women participating in both the gender dialogue group and the economic empowerment programme were less likely to report economic abuse (OR 0.39, 95% CI 0.25, 0.6, p<0.0001), and were less likely to be accepting of the justification given for violent acts (β = -0.97, 95% CI -1.67, -0.28, p = 0.006).[38] A second randomised-controlled trial conducted in the Ivory Coast examined the effect of a sixteen-week intimate partner violence prevention programme. Men who received the intervention reported a decreased intention to use physical intimate partner violence (aRR 0.83, 95% CI 0.66, 1.06). Men in the intervention arm reported a greater ability to control their hostility and manage conflict (aRR 1.3, 95% CI 1.06, 1.58). A non-statistically significant difference was identified in reported levels of physical and/or sexual intimate partner violence (aRR 0.52, 95% CI 0.18, 1.51).[39]

A third randomised controlled trial conducted in the Democratic Republic of the Congo compared the impact of either cognitive processing therapy (comprising one individual session and 11 group sessions), or individual support for female survivors of sexual violence with high levels of post-traumatic stress and combined depression and anxiety symptoms. Both the individual support and group therapy arms reported a statistically significant improvement in all checklist scores (HSCL-25 score, PSTD checklist, and the functional-impairment score) at the end of the intervention, which was sustained at six months post-intervention (p<0.001).[40]

### Interventions targeting outcomes across multiple SRH domains

Four studies reported on outcomes that spanned both family planning and pregnancy and maternal and neonatal health outcomes. A three-tiered community-based intervention in Myanmar employed traditional birth attendants to provide antenatal services and assist with deliveries, while health workers provided antenatal care and family planning supplies, and maternal health workers oversaw the aforementioned activities and attended both normal and complicated deliveries. Use of a modern method of contraception increased from 23.9% to 45.0% (prevalence rate ratio (PRR) 1.88, 95% CI 1.63, 2.17). Unmet family planning needs dropped from 61.7% to 40.5% (PRR 0.65, 95% CI 0.60, 0.72), while birth attendance by someone trained in emergency obstetric care increased from 5.1% to 48.7% (PRR 9.55, 95% CI 7.21, 12.64).[42] A cross-sectional study reviewed the possible impact of a reproductive health literacy programme amongst refugee women in Guinea. Half of the survey respondents (50%) reported current use of modern contraceptives, while 24% reported using a condom the last time they had sex, of which both findings were interpreted by study authors as an increase since implementation of the reproductive health literacy programme. The study found that 92% of women who reported becoming pregnant since the implementation of the reproductive health literacy programme reported attending at least three antenatal visits.[41]

In another study evaluating the effectiveness of an emergency obstetric care (EmOC) intervention, a trained physician was posted to a district general hospital in Makeni, Sierra Leone. Additional training was conducted, an unused operating theatre was operationalised, and a generator and blood bank were installed. The proportion of women accessing the hospital increased from 31 in 1990 to 98 in 1995, with a reduction in the case fatality rate from 32% to 5%. Notably, 444 induced abortion-related procedures were performed, compared with only 22 in 1990.[53]

Following the 2005 earthquake in Pakistan, a four-year project was initiated by USAID with the aim of improving the management capacity of district health authorities, improving access to primary healthcare services, increasing community participation in service management, and improving household-level knowledge and health-seeking behaviour. The study found non-statistically significant increases in the use of modern contraceptives (18% to 22%), at least one antenatal care visit (70.3% to 73.6%), and presence of a skilled birth attendant (36% to 38%). The study also found a statistically significant increase from 25% to 33.3% (p<0.01) in women receiving postnatal care.[43]

## Discussion

This review identified 29 studies assessing the effectiveness of SRH interventions in humanitarian crises published between 1980 and 2017. We found high quality evidence for specific interventions appearing to have improved SRH outcomes, including home visits and peer-led educational and counselling, training of lower-level health care providers, the use of CHWs to promote SRH services, a three-tiered network of health workers providing SRH services, the integration of HIV and SRH services, and men’s discussion groups to address intimate partner violence. We found moderate quality evidence to support transport-based referral systems, community-based SRH education, CHW delivery of injectable contraceptives, wider literacy programmes, and birth preparedness interventions. We identified no studies focusing on adolescents, LGBTQ populations or people with disabilities. Additionally, no studies measured the effectiveness of interventions addressing the comprehensive clinical management of rape, or the response to, or prevention of, vaginal injuries and fistulae.

The quality of studies included in the review was variable. Three quarter of the studies (72.4%, n = 21) used either experimental designs, i.e. randomised controlled trials, or quasi-experimental study designs, which provided some statistical measure of difference between intervention and outcome. However, where appropriate, there was often insufficient adjustment for potential confounders Evidence of attribution was particularly weak, with the vast majority of studies using cross-sectional and pre-post study designs with no control.

Although the majority of studies (72.4%, n = 21) were graded as high quality, the application of the STROBE checklist revealed a number of commonly recurring methodological weaknesses. Blinding was rarely used, including in the randomised controlled trials, which risks reporting bias of health outcomes. There was a lack of controlled studies, limited appreciation of clear exposures and confounders, absence of reporting on sampling methods, and an inadequate handling of bias. However, it is recognised that for a number of SRH interventions and in certain humanitarian contexts, blinding or use of control groups is not possible or appropriate. There was also limited use of stratification, for example by gender or age, and so potentially differing health outcomes in more vulnerable groups is missing.

We recognise that there are many logistical and ethical challenges related to SRH research in humanitarian crises. However, the more developed evidence base for other health outcomes in similar crises settings, e.g. mental health and communicable diseases,[16] signals that conducting more rigorous research in humanitarian contexts is possible. Given the lack of longitudinal data or studies with an adequate control comparison group, innovative ways of collecting data, e.g. using information and communication technologies (ICT) widely used by many conflict-affected populations such as WhatsApp, should be tested. These data collection methods may prove beneficial for researchers, healthcare providers, and organisations seeking to collect health outcome data at the individual-level, and from populations on the move who have traditionally been challenging to follow up. There is also a need for innovation in establishing stronger referral and follow-up systems in crisis settings to ensure health outcomes used to assess effectiveness are as close to its true effectiveness as possible. Researchers should also consider use of alternative study designs where standard RCTs are not operationally or ethically possible. Adjusted approaches such as stepped wedge trial designs could be used more widely in order to establish a counterfactual through the use of a control group, while remaining operationally and ethically acceptable. Additionally, no studies reported measuring outcomes beyond the study period, signalling the need to have data on longer-term effects of SRH interventions on target populations affected by humanitarian crises. Despite a well-established SRH evidence base in stable settings, further robust research is needed to determine effective and cost-effective interventions to improve SRH outcomes in populations in crisis settings.

This systematic review had a number of limitations. Caution must be exercised in generalising our study’s findings, as the specific contexts, and enabling and restrictive factors to intervention delivery and effectiveness, varied widely between studies. Although we based search terms and developed inclusion criteria to best address our research aim, our search may have missed identifying additional papers from relevant SRH domains. We also did not conduct a search for grey literature. Our strict study design criteria also excluded qualitative studies, which form a key component of the literature in this field. However, we only included quantitative studies demonstrating quantitative change over time with the aim of extracting a certain type of evidence to inform our findings on the effectiveness of SRH interventions in crisis settings. Additionally, only papers written in English and French were included. We applied a narrative synthesis approach to the findings, as we were unable to conduct a meta-analysis due to the heterogeneous nature of intervention types, indicators and methods used in the included studies. We used the STROBE checklist to assess the quality of reporting in studies, but a more specialised quality assessment tool such as the Newcastle Ottawa Scale or Cochrane Collaboration's tool for assessing risk of bias would have provided a more robust review of quality.[54]

This systematic review found high quality evidence to support improved SRH outcomes through home visits and peer-led educational and counselling, training of lower-level health care providers, CHWs to promote SRH services, a three-tiered network of health workers providing SRH services, the integration of HIV and SRH services, and men’s discussion groups for the reduction of intimate partner violence. However, the types of study design including many studies with no control group, the limited use of statistical data, and the variable quality of studies, signal that caution must be exercised in the interpretation of these results. There is a need for a higher quantity and quality of more timely research on the effectiveness of SRH interventions in humanitarian crises, in particular focusing on outcomes amongst adolescents, and studies that focus on safe abortion care, post-abortion care, vaginal injury and fistulae, the prevention of SGBV, and the comprehensive clinical management of rape.

## Supporting information

S1 Appendix PRISMA checklist. (DOC)

S2 Appendix Systematic review protocol. (DOCX)
