# Supplementary material for: Evaluating the effectiveness of sexual and reproductive health services during humanitarian crises: A systematic review
Source: PLoS One. 2018 Jul 6;13(7):e0199300. doi: 10.1371/journal.pone.0199300 (PMC6035047; doi:10.1371/journal.pone.0199300)
Supplement: S2 Appendix — (DOCX) [file pone.0199300.s002.docx]

# **S1 Appendix**. Systematic review protocol

# Systematic literature review on the effectiveness of sexual and reproductive health (SRH) interventions including the Minimum Initial Service Package (MISP) in humanitarian crises settings

## **1. Aim**

We aim to conduct a systematic review to address the following objective: *what is the evidence base for providing SRH interventions including the MISP from the onset of emergencies?*

Specifically, we will focus on examining the evidence base for the effectiveness of providing the MISP overall and its four pillars, abortion and adolescent-focused SRH interventions, and linking these components to a range of health outcomes (i.e. changes in unmet need for FP and abortion care, contraceptive prevalence rate, gender-based violence, skilled attendance at birth, HIV incidence, sexually transmitted infection (STI) diagnosis, and adolescent, maternal and neonatal mortality and morbidity).

## **2. Methods**

This systematic literature review aims to provide a situational analysis of the existing evidence on the effectiveness and utilisation of the Minimum Initial Service Package (MISP) in humanitarian crises settings from January 1, 1980 to April 9, 2017. It will provide an update to an earlier systematic review on the effectiveness of the MISP conducted by Warren et al. (2015) that reviewed peer-reviewed literature from January 1, 1980 to April 30, 2013. [1]. The systematic review methodology will adhere to the Preferred Reporting Items for Systematic Reviews and Meta-Analyses (PRISMA) statement. [2]

### *2.1 Key terms:*

The following key terms and concepts relate to this systematic literature review, their definitions having been adapted from the World Health Organization (WHO) Humanitarian Health Action Dictionary. [3]

Public Health Intervention: Public health actions that seek to improve health outcomes.

Humanitarian crisis: A serious disruption of the functioning of a community or a society causing widespread human, material, economic or environmental losses which exceed the ability of the affected community or society to cope using its own resources, necessitating a request to national or international level for external assistance. The disaster situation may be either manmade (e.g. armed conflict) or a natural phenomenon (e.g. drought).

Man-made humanitarian disasters: These include international armed conflicts; non-international armed conflicts; and other situations of violence. [4]

Natural disasters: These include hazardous natural phenomena leading to humanitarian crises such as earthquakes; volcanic activity; landslides; tsunamis; tropical cyclones and other severe storms; tornadoes and high winds; floods, and droughts.

Early Recovery: Early Recovery is defined as recovery that begins early in a humanitarian setting. It is a multi-dimensional process, guided by development principles. It aims to generate self-sustaining nationally owned and resilient processes for post-crisis recovery.

### *2.2 Search strategy and search terms*

This literature review will use peer-reviewed literature, which will be located using the following electronic bibliographic databases: Medline, Embase, Global Health, PsychINFO, International Bibliography of the Social Sciences (IBSS), and Web of Science. The search structure will consist of the following:

- terms related to humanitarian crises;
- AND terms related to public health interventions;
- AND terms related to lower and middle income economies;
- AND terms related to SRH interventions including the MISP

The search terms used for the main bibliographic databases are given in Section 2. The additional specific search terms related to the MISP will then be added on (see Section 3).

Searches were supplemented by reviewing the reference lists (‘references of references’) of selected articles to find any other relevant papers.

### *2.3 Inclusion/exclusion criteria*

The following seven key inclusion criteria will be used in this review:

- Types of studies: Primary quantitative research studies. Study designs including randomised controlled trials, non-randomised controlled trials, controlled before-after studies, controlled interrupted time series studies, economic studies (cost-effectiveness analysis, cost-utility analysis, cost-benefit analysis, economic modelling) of public health which the outcome is measured before and after the intervention or an intervention is studied against another intervention with baseline or control group.
- Populations of interest: Populations affected by humanitarian crises and receiving humanitarian assistance in low and middle-income countries (based upon World Bank country classification).
- Health outcomes of interest: Primary outcomes include adolescent, maternal and neonatal morbidity; adolescent, maternal and neonatal mortality; STI diagnosis; gender-based violence; and unmet need for family planning. Secondary outcomes include contraceptive prevalence rate; skilled attendance at birth; and emergency obstetric and newborn care (EmONC)
- Crisis Phase: Studies that occur in humanitarian crises including those that evaluate: i) the impact of preparedness and resilience on public health outcomes during a humanitarian crises and/or ii) studies that evaluate the impact of public health interventions during the acute, chronic, or early recovery phases of humanitarian crises.
- Data type(s): Must include primary data.
- Date of intervention and publication: May 1, 2013 – April 10, 2017.
- Publication language: English, French.

The following criteria will be used to exclude studies from this review:

- Studies with no specific health intervention and no outcomes (i.e., excluding studies that examine only health needs, prevalence, health risk-factors, co-ordination).
- Studies that examine preparedness and resilience not linked to health outcomes in humanitarian crises (e.g. studies on housing fortification before flooding).
- Review papers; only references listed in review papers will be screened to find more primary data sources.

### *2.4 Study screening and data extraction*

The systematic literature review will be conducted by two reviewers for quality assurance.

Data will be screened with the following five stages:

*Stage One*: electronic database search using terms; with results imported into reference management software, and duplicates removed.

*Stage Two*: title and abstract reviewed to remove studies not meeting the inclusion criteria (see above).

*Stage Three:* manuscript review to remove studies that did not meet inclusion criteria; paper selection.

*Stage Four:* review of references of selected papers (from Stage Three).

*Stage Five:* final paper selection, data extraction, and quality assessment.

Data will be extracted based on the specific points noted below and inputted into a standardised Excel database:

- study authors or agency, year
- study country
- setting: urban or rural
- population type (refugee; internally displaced; entrapped population; host population)
- humanitarian crises type (armed conflict or natural disaster)
- health outcome(s) addressed by the public health intervention
- type(s) of public health intervention
- study design
- measurement outcomes (e.g. prevalence, odds ratios etc)
- quality of the evidence on specific interventions
- change in quantity of evidence over time
- change in quality of evidence over time
- research strengths from the literature
- research gaps from the literature

### *2.5 Data categorisation and analysis*

Data findings will be organised in relation to the key issues of quantity and quality of the evidence base. To increase clarity of the final results, the studies selected at Stage Five will be arranged into three main categories of evidence (**Table 1**):

**Table 1: Categorisation of selected literature**

| **Category A:**  Studies that measure statistical associations between intervention and health-related outcome |
| --- |
| **Category B:**  Studies that measure changes in health-related outcome, but do not report statistical associations. |
| **Category C:**  Outcomes not measured (e.g. outputs, processes, perceptions) |

As indicated in **Table 1**, Categories A and B roughly correspond to evidence that is expected to be of high to moderate quality. Given the generally much weaker value of evidence in Category C, data extracted from studies classified as Category C was limited to the existence of the study alone.

The quality assessment of studies (Categories A and B) that will be included in the systematic literature review will be reviewed based upon criteria adapted from the STROBE and CONSORT standards for observational studies and clinical trials, respectively. The adaptations are outlined in

Table 2 and scoring levels given in Table 3.

**Table 2: Quality review criteria (adapted from STROBE and CONSORT)**

| **STROBE Criteria for Observational Studies*** | **CONSORT Criteria for Clinical Trials*** |
| --- | --- |
| Intervention:  1. Is the intervention clearly described?  Selection of participants:  2. Is the target population defined?  3. Is there a comparison group (e.g. baseline, control)?  4. Are the inclusion and exclusion criteria defined?  Statistical methods:  5. Is the sample size / method justified with statistical basis?  6. Is there a statistical test (p-value or confidence interval)?  7. Is there adjustment for confounding?  Limitations:  8. Are study limitations explained (e.g. biases)? | Eligibility  1. Did study state # not meeting inclusion criteria?  2. Did study state # declined to participate?  Once Randomized:  Allocation:  3. Did study state # receiving intervention?  4. Did study state # not receiving intervention?  Follow-Up:  5. Did study state # lost to follow-up?  6. Did study provide reasons for loss to follow-up?  Analysis:   1. Did study state reasons participants were excluded from analysis? 2. Are limitations of the study explained (e.g. biases) |

**Table 3: Quality assessment corresponding to adapted STROBE and CONSORT criteria**

| **Level of Quality** | **Rating of Evidence per STROBE / CONSORT** |
| --- | --- |
| HIGH | 7-8 criteria met = **high** quality evidence |
| MODERATE | 4-6 criteria met = **moderate** quality evidence |
| LOW | 1-3 criteria met = **low** quality evidence |

## **3. Search terms used for key bibliographic databases**

| 1 | exp Disasters |
| --- | --- |
| 2 | exp Relief Work |
| 3 | Rescue Work |
| 4 | Emergencies |
| 5 | Emergency Medicine |
| 6 | Emergency Medical Services |
| 7 | Disaster Medicine |
| 8 | Mass Casualty Incidents |
| 9 | Emergency Responders |
| 10 | Medical Missions, Official |
| 11 | (humanitarian adj2 (crisis or crises or relief or response or agenc$)).tw. |
| 12 | humanitarian.tw. |
| 13 | (disaster adj3 (relief or plan$)).tw. |
| 14 | ((relief or aid) adj2 work$).tw. |
| 15 | Refugees |
| 16 | (refugee or evacuee or evacuated).tw. |
| 17 | (displace$ adj2 (force$ or population or human or internal$)).tw. |
| 18 | Altruism |
| 19 | exp War |
| 20 | war.tw. |
| 21 | ((armed or zone) adj2 conflict$).tw. |
| 22 | (conflict affected adj3 (population$ or person$ or communit$)).tw. |
| 23 | Avalanches |
| 24 | Earthquakes |
| 25 | Floods |
| 26 | Landslide |
| 27 | Tidal Waves |
| 28 | Tsunamis |
| 29 | Cyclonic Storms |
| 30 | (typhoon$ or hurricane$ or cyclone$).tw. |
| 31 | (avalanche$ or earthquake$ or flood or floods or flooding or flooded or landslide$ or tsunami$).tw. |
| 32 | (disaster adj2 (natural or victim)).tw. |
| 33 | Droughts |
| 34 | drought$.tw. |
| 35 | Starvation |
| 36 | (starvation or famine$).tw. |
| 37 | or/1-36 |
| 38 | randomized controlled trial |
| 39 | controlled clinical trial |
| 40 | cross-sectional studies |
| 41 | case-control studies |
| 42 | cohort studies |
| 43 | pilot studies |
| 44 | (random$ or controlled).tw. |
| 45 | (control adj3 (area or cohort? or compare? or condition or design or group? or intervention? or participant? or study)).ab. not (controlled clinical trial or randomized controlled trial).pt. |
| 46 | ((evaluat$ or prospective or retrospective) adj1 study).tw. |
| 47 | ("quasi-experiment$" or quasiexperiment$ or "quasi random$" or quasirandom$ or "quasi control$" or quasicontrol$ or ((quasi$ or experimental) adj3 (method$ or study or trial or design$))).tw. |
| 48 | ("time series" adj2 interrupt$).tw. |
| 49 | (intervention$ or impact or effectiveness or efficacy or service$ or outcome$ or output or treatment$ or management or program$ or project$).tw. |
| 50 | economics |
| 51 | cost-benefit analysis |
| 52 | cost control |
| 53 | Cost savings |
| 54 | cost of illness |
| 55 | cost $utility.tw. |
| 56 | (Cost$ adj2 effective$).tw. |
| 57 | cost-effective$.tw. |
| 58 | (cost adj3 utility).tw. |
| 59 | cost-utilit$.tw. |
| 60 | or/38-59 |
| 61 | developing countries |
| 62 | exp asia |
| 63 | exp africa |
| 64 | exp pacific islands |
| 65 | exp eastern europe |
| 66 | exp china |
| 67 | balkan peninsula/ or europe, eastern/ or transcaucasia |
| 68 | caribbean region/ or central america/ or "gulf of mexico"/ or latin america/ or south america |
| 69 | atlantic islands/ or indian ocean islands/ or macau/ or pacific islands/ or philippines/ or prince edward island/ or svalbard/ or west indies |
| 70 | or/61-69 |
| 71 | Japan |
| 72 | 70 not 71 |
| 73 | 37 and 60 and 72 |
| 74 | limit 73 to yr="2013 -2017” |

**4. Search terms specific to the Effectiveness of SRH interventions including the MISP**

***Sources:***

Published literature: Embase, Global Health, Medline, International Bibliography of the Social Sciences (IBSS), PsychINFO and Web of Science.

**Sexual and Reproductive Health**

"Reproductive Health"[Mesh] OR "Reproductive Medicine"[Mesh] OR "Sexual Dysfunction, Physiological"[Mesh] OR "Sexual Dysfunctions, Psychological"[Mesh] OR "Libido"[Mesh] OR "Sexology"[Mesh] OR "Sex Education"[Mesh] OR "Sexual Behavior"[Mesh] OR "Sexual Behaviour"[Mesh] OR "Sexuality"[Mesh] OR "Sexual Partners"[Mesh] OR "Sexual Minorities"[Mesh] OR "Sex Workers"[Mesh] OR “Sexual Health”[TIAB] OR “sexual health”[Mesh] OR “Sexuality”[TIAB] OR “Sexual Dysfunction”[TIAB] OR “Libido”[TIAB] OR “Sex Education”[TIAB] OR “Sexuality Education”[TIAB] OR “Sexology”[TIAB] OR “Sex Counselling”[TIAB] OR “Sex Counseling” [TIAB] OR “Sexual Behavio#r” [TIAB] OR “Sexual Partner*”[TIAB] OR “Sexual Minorit*”[TIAB] OR “Minimum Initial Service Package”[TIAB]

**Contraception**

“Reproductive plan*”[tiab] OR “contracepti*”[tiab] OR “contraceptive use”[tiab] OR “birth control”[tiab] OR “birth spacing”[tiab] OR “child spacing”[tiab] OR “condom*”[tiab] OR “the pill”[tiab] OR “oral contraceptive”[tiab] OR “oral contraceptives”[tiab] OR “microbicide”[tiab] OR “diaphragm”[tiab] OR “IUD”[tiab] OR “Intrauterine device”[TiAB] OR "Intrauterine Devices, Medicated"[Mesh] OR "Intrauterine Devices, Copper"[Mesh] OR  "Intrauterine Devices"[Mesh] OR “Drug implant*”[MeSH] OR “Drug implant*”[tiab] OR “Drug Pellet*”[tiab] OR “Levonorgestrel”[MeSH] OR “Norethindrone”[MeSH] OR “contraceptive implant*”[tiab] OR “progestogen only contraceptive*”[tiab] OR “progestogen implant*”[tiab] OR “etonogestrel implants”[tiab] OR “Implanon”[tiab] OR “Subdermal contraceptive implant*”[tiab] OR “Norplant” [tiab] OR “Jadelle”[tiab] OR “Sino-implant”[tiab] OR “Depo Provera”[tiab] OR “Nexplanon”[tiab] OR “Norprogesterones”[tiab] OR “natural family planning”[tiab] OR “lactational amenorrhea”[tiab] OR OR “LAM”[tiab]” OR “postpartum amenorrhea”[tiab] OR “post-partum amenorrhea”[tiab] OR “amenorrhea”[mesh] OR “periodic abstinence”[tiab] OR “rhythm method”[tiab] OR “calendar method”[tiab] OR “sexual abstinence”[tiab] OR “Family Planning Services”[MeSH] OR “Reproductive Health Services”[MeSH] OR “Reproductive Health Services”[tiab] OR “Reproductive Medicine”[MeSH] OR “Contraception”[MeSH] OR “Contraception, Postcoital”[MeSH] OR “Contraception, Immunologic”[MeSH] OR “Contraception, Barrier”[MeSH] OR “Contraception Behaviour”[MeSH] OR “Natural Family Planning Methods”[MeSH] OR “Contraceptive Devices, Female[MeSH]” OR “Contraceptive Agents, Female”[MeSH] OR “Condoms, Female”[MeSH] OR “Sterilization, Reproductive”[MeSH] OR “family planning” [tiab] OR (“family planning” [Tiab] AND service* [TiAB]) OR (“family planning” [Tiab] AND program* [TiAB]) OR “Family Planning Services” [MeSH] OR “Family Planning Service”[tiab] OR “Family Planning”[MeSH] OR  “Family Planning Programs”[Mesh] OR “Family Planning Program”[tiab] OR “population control”[mesh]

NOT Animals [mesh] NOT “Animal experimentation” [Mesh] NOT “Models, Animal” [Mesh]

**Emergency Contraception:**

"Contraceptives, Oral/administration and dosage"[Mesh] OR "Contraceptives, Oral/metabolism"[Mesh] OR "Contraceptives, Oral/pharmacology"[Mesh] OR "Contraceptives, Oral/physiology"[Mesh] OR "Contraceptives, Oral/therapeutic use"[Mesh]) AND "Contraceptives, Postcoital/administration and dosage"[Mesh] OR "Contraceptives, Postcoital/adverse effects"[Mesh] OR "Contraceptives, Postcoital/contraindications"[Mesh] OR "Contraceptives, Postcoital/metabolism"[Mesh] OR "Contraceptives, Postcoital/pharmacokinetics"[Mesh] OR "Contraceptives, Postcoital/standards"[Mesh] OR "Contraceptives, Postcoital/supply and distribution"[Mesh] OR "Contraceptives, Postcoital/therapeutic use"[Mesh] OR "ulipristal acetate"[Supplementary Concept]

**Medical abortion**

"Abortion, Induced"[Mesh] OR "Abortion, Incomplete"[Mesh] OR "Abortion, Spontaneous"[Mesh] OR “abortion” [tiab] OR “miscarriage”[tiab] OR “pregnancy termination”[tiab] OR “termination of pregnancy”[tiab] OR “abortal"[Tiab] OR "postabortion"[Tiab] OR "post-abortal"[Tiab] OR “postabortion care”[tiab] OR “incomplete abortion*”[tiab] OR "Mifepristone"[Mesh] OR "Misoprostol"[Mesh] OR “RU486”[tiab] OR “mifegyne”[tiab] OR “Cytotec”[tiab] OR “Medabon”[tiab] OR “medication abortion”[tiab] OR “medical abortion”[tiab] OR "unsafe abortion"[Tiab] OR "unsafe abortions"[Tiab] OR (("aftercare"[Tiab] OR "after care"[Tiab]) OR "aftercare"[Tiab]) OR ("postoperative"[Tiab] OR "post-operative"[Tiab]) AND "abortion"[Tiab])

**Surgical abortion**

"Abortion, Induced"[Mesh] OR "Abortion, Incomplete"[Mesh] OR "Abortion, Spontaneous"[Mesh] OR “abortion” [tiab] OR “miscarriage”[tiab] OR “pregnancy termination”[tiab] OR “termination of pregnancy”[tiab] OR “postabortion care” [tiab] OR “incomplete abortion”[tiab] OR "Extraction, Obstetrical"[Mesh] OR "Dilatation and Curettage"[Mesh] OR "Vacuum Curettage"[Mesh] OR “surgical abortion” [tiab] OR “dilation and evacuation”[tiab] OR “D&E”[tiab] OR “suction curettage”[tiab] OR “vacuum aspiration”[tiab] OR “D&C”[tiab] OR “menstrual regulation”[tiab]

**Abortion-related complications**

"Hemorrhage"[Mesh] OR "Postoperative Hemorrhage"[Mesh] OR "Uterine Hemorrhage"[Mesh] OR "Postpartum Hemorrhage"[Mesh] OR "Infection"[Mesh] OR "Pelvic Infection"[Mesh] OR "Uterine Perforation"[Mesh] OR "Uterine Rupture"[Mesh] OR "Pregnancy Complications"[Mesh] OR "Postoperative Complications"[Mesh] OR "Intraoperative Complications"[Mesh] OR "Emergency Treatment"[Mesh] OR “Abortion, Septic” [Mesh] OR “haemorrhage” [tiab] OR “haemorrhage”[tiab] OR “hemorrhage”[tiab] OR “bleeding”[tiab] OR “endometritis”[tiab] OR “parametritis”[tiab] OR “metritis” [tiab] OR “pelvic infection” [tiab] OR “uterine infection” [tiab] OR “uterine perforation” [tiab] OR “abortion-related complications”[tiab] OR “emergency care” [tiab] OR “ongoing pregnancy” [tiab] OR “ectopic pregnancy”[tiab] OR “emergency treatment” [tiab] OR “EmOC”[tiab] OR “emergency obstetric care”[tiab] OR “complications”[tiab] or “stillb*”[tiab] OR “birth”[tiab]

**HIV/AIDS**

“HIV”[Mesh] OR “AIDS”[tiab] OR “HIV/AIDS”[tiab] OR “Human Immunodeficiency Virus”[Mesh] OR “Human Immune Deficiency Virus”[tiab] OR “Acquired Immunodeficiency Syndrome”[Mesh] OR “Acquired Immune Deficiency Syndrome”[tiab] OR HIV [MesH] OR HIV-1 [MesH] OR HIV-2 [MesH] OR “HIV infections” [MesH] OR “acquired immunodeficiency syndrome” [MesH] OR “HIV seropositivity” [MesH] OR “HIV seroprevalence” [MesH] OR “AIDS serodiagnosis” [MesH]

**STIs**

“Chlamydia infections”[Mesh] OR “Gonorrhea”[Mesh] OR “chlamydia”[tiab] OR “gonorrhoea”[tiab] OR “syphilis”[tiab] OR “syphilis”[Mesh] OR “sexually transmitted infections”[Mesh] OR “sexually transmitted infection$”[tiab] OR “sexually transmitted disease$”[tiab] OR “sexually transmitted disease$”[Mesh] OR “hepatitis”[tiab] OR “chancroid”[tiab] OR “trichomoniasis”[tiab] OR “human papillomavirus”[tiab] OR “HPV”[tiab] OR “genital wart$”[tiab] OR “herpes”[tiab] OR “bacterial vaginosis”[tiab] OR “scabies”[tiab] OR “public lice”[tiab] OR “crab lice”[tiab] OR “pelvic inflammatory disease”[tiab] OR “PID”[tiab] OR “mucopurulent cervicitis”[tiab] OR “MPC”[tiab] OR “molluscum contagiosum”[tiab] OR “lymphogranuloma venereum”[tiab] OR “LGV”[tiab]

**PMTCT**

“antenatal HIV test”[tiab] OR “maternal HIV test”[tiab] OR “infant HIV test”[tiab] OR “infant HIV diagnosis”[tiab] OR “option A”[tiab] OR “option B”[tiab] OR “option B+”[tiab] OR “nevirapine”[tiab] OR “mother-to-child transmission”[tiab] OR “maternal transmission”[Mesh] “maternal transmission”[tiab] OR MTCT[tiab] OR “prevention of mother-to-child transmission”[tiab] OR “PMTCT”[tiab] OR “PMTCT cascade”[tiab]

**Pregnancy, Maternal and Newborn Health**

“Pregnan*"[Mesh] OR "Pregnancy, Unplanned"[Mesh] OR “"Pregnancy, unwanted"[Mesh] OR “Pregnancy in adolescence”[Mesh] OR “Pregnancy outcome”[Mesh] OR “Pregnancy, complication*”[Mesh] OR “Pregnan*”[tiab] OR “IUP”[tiab] OR “Intrauterine pregnancy”[tiab] “Pregnancy, complication*”[tiab] OR “Maternal health” [Mesh] OR “Maternal health” [tiab] OR “Maternal welfare” [Mesh] OR “Maternal welfare” [tiab]OR “Safe motherhood”[Mesh] OR “Safe motherhood”[tiab] OR “Perinatal”[Mesh] OR

“Perinatal”[tiab] OR “Perinatal care”[Mesh] OR “Perinatal health”[Mesh] OR “Prenatal care”[Mesh] OR “Prenatal health”[Mesh] OR “Prenatal diagnosis”[mesh] OR “Perinatal care”[tiab] OR “Perinatal health”[tiab]

OR “Postnatal health”[Mesh] OR “Antenat*”[Mesh] OR “Antenatal health”[Mesh] OR “Antenatal*”[tiab] OR “Ante-natal*”[tiab] OR “Prenatal*”[Mesh] OR “Prenatal*”[tiab]  OR “postnatal care”[tiab] OR “postnatal”[tiab] OR “post-natal”[tiab] OR “Postpart*”[Mesh] OR “Post-part*” [Mesh] OR “Parturition”[Mesh] OR “Postpartum period”[MeSH] OR “Postpartum”[tiab] OR “Post-partum”[tiab] OR “Puerperium”[tiab] OR “Gestation”[tiab] OR “Postbirth”[tiab] OR “Post-birth”[tiab] OR “Mother*”[Mesh] OR “Matern*”[Mesh] OR “Childbirth”[tiab] or “Obstetrics”[tiab] OR “obstetric*”[Mesh] OR “Gynecology”[Mesh] OR “Labor pain”[Mesh] OR “Analgesia, Obstetric” [Mesh] OR “Obstetric Surgical Procedures” [Mesh] OR “Delivery, Obstetric”[Mesh] OR “Safe delivery”[Mesh] OR “Safe delivery”[tiab] OR “Skilled birth attend*”[Mesh] OR “Skilled birth attend*”[tiab] OR “Maternal Health Service*” [Mesh] OR “Emergency Obstetric Care”[tiab] OR “Emergency Obstetric and Newborn Care”[tiab] OR “EmOC”[tiab] OR “EmONC”[tiab] OR “Infant”[Mesh] OR “Neonat*”[Mesh] “Neonatal health”[Mesh] OR “Infant health”[Mesh] or “Newborn health”[Mesh] OR “Newborn infant health”[mesh] OR “Infant welfare”[Mesh] OR “Baby health”[Mesh] OR “Newborn”[Mesh] OR “Infant, Newborn” [Mesh] OR “Birth*”[Mesh] OR “Fetus”[mesh] OR “Fetal therapies”[mesh] OR “Fetal monitoring”[mesh] OR “Infant”[tiab] OR “Neonat*”[tiab] “Neonatal health”[tiab] OR “Infant health”[tiab] OR “newborn health”[tiab] OR “Newborn infant health”[tiab] OR “Infant welfare”[tiab] OR “Baby health”[tiab] OR “Newborn”[tiab]  OR “Stillbirth”[Mesh] OR “Still-birth”[Mesh] OR “Stillbirth”[tiab] OR “Still-birth”[tiab]

**Vaginal Injury & Fistulas**

“fistula”[Mesh] OR “fistula”[tiab] OR “rectovaginal fistula”[tiab] OR “rectovaginal fistula”[Mesh] OR “vaginal fistula”[Mesh] OR “urethra fistula”[tiab] OR “urinary tract fistula”[tiab] OR “genital trauma”[tiab] or “genital injury”[tiab] or “vaginal trauma”[tiab] or “vaginal injury”[tiab]

**Adolescent Health**

“adolescent sexual health”[tiab] OR “adolescent reproductive health”[tiab]OR “adolescent health”[tiab] OR “adolescent health services”[tiab] OR “youth friendly services”[tiab] OR “adolescent friendly services”[tiab] OR “adolescent health service”[tiab] OR “youth friendly service”[tiab] OR “adolescent friendly service”[tiab] OR “youth program*”[tiab] OR “pregnant adolescents” [Mesh]

**Gender-based violence**

“gender-based violence”[tiab] OR “partner violence”[tiab] OR “family violence”[tiab] OR “violence against women”[tiab] OR “domestic violence”[tiab] OR “sexual abuse”[Mesh] OR “sexual abuse”[tiab] OR “sex crime”[tiab] OR “sexual crime”[tiab] OR “domestic violence”[tiab] OR “domestic violence”[Mesh] OR “family violence”[Mesh] or “sexual violence”[tiab] OR “sexual violence”[Mesh] OR “rape”[tiab] OR “physical violence”[tiab] OR “rape”[Mesh] OR “intimate partner violence”[tiab] OR “intimate partner violence”[Mesh] OR “partner violence”[tiab] OR “partner abuse”[tiab] OR “spousal abuse”[tiab] OR “spouse abuse”[Mesh] OR “wife abuse”[tiab] OR “partner violence”[Mesh] OR “assault”[tiab] OR “physical assault”[tiab] OR “sexual assault”[tiab] OR “sexual crime”[tiab] OR “sexual harassment”[Mesh] OR “sexual harassment”[tiab] OR “sexual coercion”[tiab] OR “forced sex”[tiab] OR “sexual slavery”[tiab] OR “abused woman”[tiab] OR “abused women”[tiab] OR “battered woman”[tiab] OR “battered women”[tiab] OR “battered women”[Mesh] OR “woman, abused”[tiab] OR “woman, battered”[tiab] OR “women, abused”[tiab] OR “women, battered”[tiab]
